# Applications, challenges, and needs for employing synthetic biology beyond the lab

**DOI:** 10.1038/s41467-021-21740-0

**Published:** 2021-03-02

**Authors:** Sierra M. Brooks, Hal S. Alper

**Affiliations:** 1 McKetta Department of Chemical Engineering, The University of Texas at Austin, Austin, TX USA; 2 Institute for Cellular and Molecular Biology, The University of Texas at Austin, Austin, TX USA

**Keywords:** Biotechnology, Synthetic biology

## Abstract

Synthetic biology holds great promise for addressing global needs. However, most current developments are not immediately translatable to ‘outside-the-lab’ scenarios that differ from controlled laboratory settings. Challenges include enabling long-term storage stability as well as operating in resource-limited and off-the-grid scenarios using autonomous function. Here we analyze recent advances in developing synthetic biological platforms for outside-the-lab scenarios with a focus on three major application spaces: bioproduction, biosensing, and closed-loop therapeutic and probiotic delivery. Across the Perspective, we highlight recent advances, areas for further development, possibilities for future applications, and the needs for innovation at the interface of other disciplines.

## Abstract (web summary)

Current developments in synthetic biology are not immediately applicable outside of the controlled laboratory environment. In this Perspective, the authors outline the advances and challenges the field faces in operating in resource limited and off-the-grid scenarios.

## Introduction

Synthetic biology and its applications hold great promise for addressing global humanitarian needs including the goals of sustainable development, zero hunger, health and well-being, reduced inequality, and improved access to responsibly produced goods and services^1^. Advances in recent years have demonstrated the potential for synthetic biology to revolutionize technologies across disparate applications including biocomputing^2,3^, living materials^4^, electronic interfacing^5^, therapeutic genome editing^6^, multiplexed diagnostics, and cellular recording^7^, third-generation biorefineries^8^, and living biotherapeutics^9^. Perhaps the most immediately recognized and advanced application of synthetic biology is the ability to alter metabolism to produce high-value products for applications ranging from biofuels and plant natural products^10,11^ to polymer precursors and bio-inspired materials^12^. In this regard, the ability to transform microorganisms into chemical factories that compete with organic chemical synthesis is ushering in a new era of biomanufacturing.

Despite these great advances, most current developments are not immediately translatable to “outside-the-lab” application spaces, which are quite diverse and variable compared to the well-controlled conditions available in laboratory settings. We posit that outside-the-lab scenarios encompass three main settings with respect to available resources: (1) resource-accessible, (2) resource-limited, and (3) off-the-grid. Resource-accessible settings include situations whereby technology is deployed with essentially unlimited access to resources and experienced personnel. Such situations (for which large-scale industrial biotechnology settings are a great example), typically entail technology transfer of lab-scale results followed by iterative process optimizations/scale-up and biological re-design cycles. However, successful deployment (even in these most resource-accessible conditions) is not a guarantee due to a number of factors including genetic stability, economics, feasibility, and other technical challenges. Resource-limited settings include scenarios marked by technology deployment in more remote settings that include more limited (but nonzero) access to resources and/or expertise, such as remote military and space missions. The most extreme condition, off-the-grid settings, include situations with minimal or no access to resources, electrical power, communication infrastructure, and expertise, such as deployment in remote areas on the planet or even within the gut microbiome. These applications necessitate that deployed technologies operate autonomously without external resources or intervention.

**Fig. 1:**
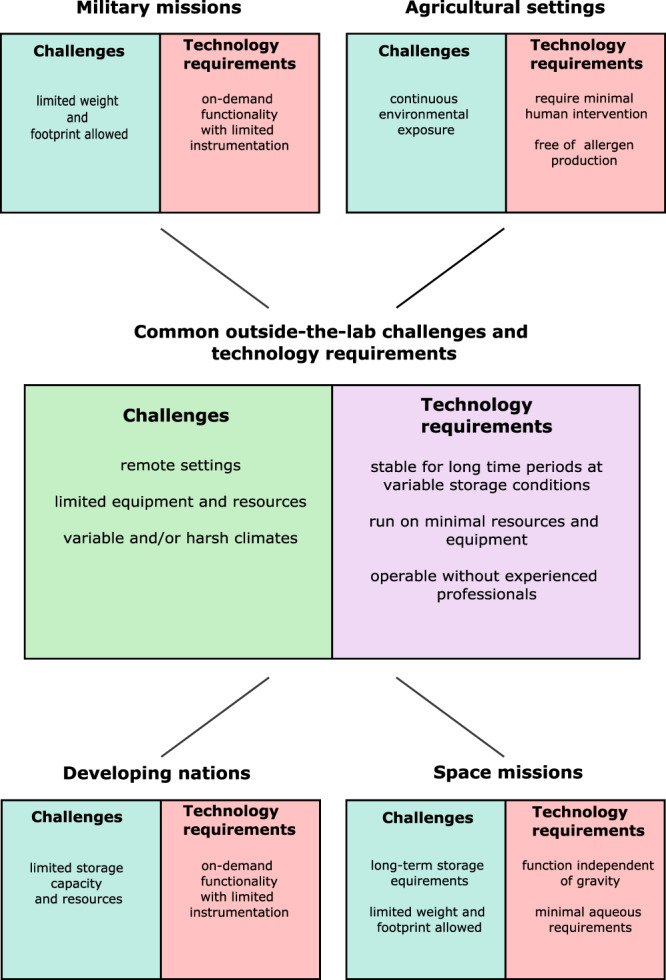
Overview of major challenges and requirements for deploying synthetic biology-based platforms in outside-the-lab settings. Outside-the-lab scenarios have wide-ranging challenges and requirements for synthetic biology that are more demanding than typical laboratory settings. This figure provides a basic overview of major challenges and requirements associated with four major outside-the-lab settings including: space missions, developing nations, military missions, and agricultural settings. Challenges and requirements common to all settings are listed in the center, and those specific to certain outside-the-lab settings are listed in their respective boxes.

As opposed to the (comparatively simpler) technology transfer and scale-up considerations typically needed in resource-accessible settings, resource-limited and off-the-grid settings require new synthetic biology paradigms to allow for successful deployment. Inherent in these applications are demanding requirements including the need for a high degree of system flexibility, long-term storage capabilities, intermittent/repeated usage, and an ability to operate with limited equipment and intervention. Many of the major challenges and technological requirements for deploying synthetic biology-based technologies in major outside-the-lab settings (specifically highlighting space missions, developing nations, military missions, and agricultural settings) are listed in Fig. 1. To be successful, outside-the-lab platforms should be genetically and functionally stable over long time periods under variable storage conditions, require minimal equipment and resources to run, and require minimal intervention by experienced professionals. In this regard, synthetic biology is undergoing a shift in paradigm from utilizing biology to deploying biology.

Both cell-based and cell-free system approaches have associated advantages and challenges with regard to ease of deployment in outside-the-lab situations. For instance, while whole-cell platforms are typically easier to mass-produce as well as consolidate multiple complex assays or reactions^13,14^, there are challenges with long-term viability/stability, toxicity of the analytes or reaction components, and time delays due to the need for cell growth and analyte/nutrient transport^15^. Cell-free platforms can address many of these associated challenges as they bypass the need for viable cells (and thus can be used to detect or produce compounds typically toxic to cells). This feature of an open reaction environment facilitates the manipulation of metabolism, transcription, and translation^16^, for instance through exogenous addition of non-native substrates. The elimination of the requirement to sustain life also confers the ability to solely focus the system’s resource utilization on a product or reaction of interest^15^. However, significant batch-to-batch variability has been demonstrated across academic labs in the context of cell-free protein synthesis yields^17^. Furthermore, the short cell-free reaction durations (typically on the order of hours)^15^, high reagent costs (particularly for energy sources and nucleotides), as well as difficulties in folding complex protein products^18^ present limitations in the application spaces for which cell-free platforms are currently viable.

We focus this Perspective on three major application spaces for the outside-the-lab deployment of synthetic biology: bioproduction, biosensing, and closed-loop living therapeutic and probiotic delivery. We open each major section with a brief intro into its applicability for outside-the-lab developments, analyze current work being done in that area, discuss areas where ongoing improvement is required to enable outside-the-lab deployment, and close with some potential scenarios to which outside-the-lab technology could be applicable.

## Production in remote and non-conventional environments

Synthetic biology has begun to enable both on-demand and continuous (or responsive) production of biochemicals, therapeutics, and even food/food-ingredients using a range of host organisms. The contributions of synthetic biology innovations toward the improved industrial production of small molecules have certainly been well-catalogued elsewhere^10,11,19–21^. In contrast, less attention has been given to production technologies applicable for outside-the-lab scenarios such as on-demand production of small molecules and proteins in developing nations, during remote military and space missions, or for other built-environment in situ production applications. Recognizing the demands and challenges associated with outside-the-lab bioproduction, a number of funding initiatives have been established including the DARPA Battlefield Medicine program aiming to overcome obstacles for on-demand manufacturing through Pharmacy on Demand (PoD) and Biologically derived Medicines on Demand (Bio-MOD) initiatives^22^. Furthermore, NASA’s Translational Research Institute for Space Health (TRISH)^23^ seeks to support astronaut health and performance on space missions, including on-demand therapeutic manufacturing on-board spacecrafts^24^.

**Fig. 2:**
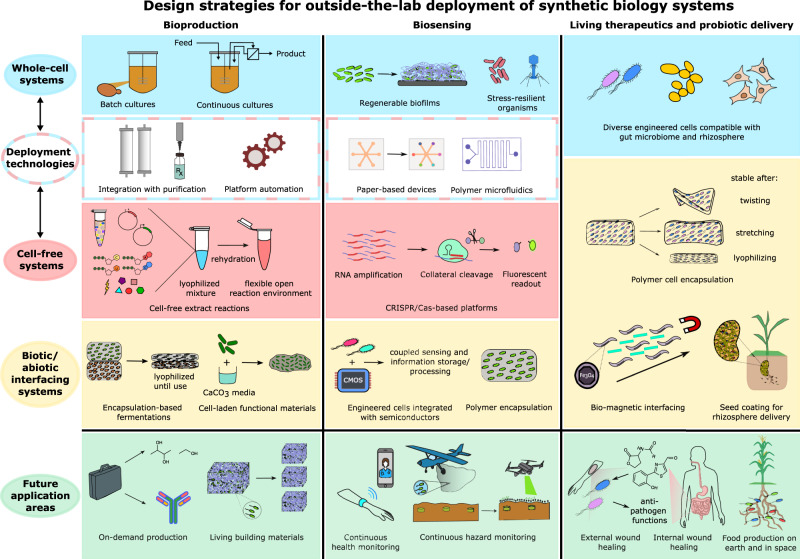
Design strategies for outside-the-lab deployment of synthetic biology systems. This Perspective encompasses design strategies for deploying synthetic biology outside-the-lab, which vary based on the particular system type (whole-cell (blue), cell-free (red), biotic/abiotic interfacing (yellow)) and application space (bioproduction, biosensing, living therapeutics, and probiotic delivery; all in green). Outside-the-lab bioproduction design strategies include whole-cell liquid cultures, cell-free extract reactions, and encapsulation platforms interfacing living cells with materials, with widespread future applications including on-demand production of small molecules and biologic therapeutics as well as regenerable living building materials. Outside-the-lab biosensing design strategies include whole-cell engineered stress-resilient organisms and regenerable biofilms, cell-free CRISPR/Cas-based sensing platforms, as well as interfacing living cells with novel polymer and electronic systems, with broad future applications including continuous health and hazard monitoring. For bioproduction and biosensing, both whole-cell and cell-free systems are typically interfaced with deployment technologies, such as platform automation and microfluidic liquid handling, to facilitate outside-the-lab usability. Outside-the-lab closed-loop living therapeutics and probiotic delivery design strategies include whole-cell engineered microbes and mammalian cells compatible with the gut and soil microbiomes, as well as interfacing living cells with materials and magnetic systems, with future applications ranging from wound healing to continuous food production on earth and in space.

Efforts using both whole-cell and cell-free technologies as well as synergistic developments with material science have demonstrated proof-of-concept studies of outside-the-lab molecule production applications. At their core, on-demand and continuous production functionalities entail the preservation and maintenance of metabolic activity in diverse settings. This requires field-deployable platforms to be genetically and environmentally stable, both while in long-term storage and metabolically active states. In addition, coupling of enhanced platform stability with user-friendly deployment technologies (such as integrated production and purification modules and liquid handling capacities) is needed for deployment in settings with limited or no access to resources or experienced personnel (Fig. 2).

### Whole-cell production platforms

Traditional cell-based recombinant production platforms have served as the primary mode of production for protein therapeutics since the development of recombinant insulin in 1982^25^ and continue to be used for the production of active pharmaceutical ingredients^26^. However, the reliance on Chinese Hamster Ovary (CHO) cell platforms for production^27^ poses issues for the development of rapid and shelf-stable on-demand production platforms given the lack of mammalian cell viability following harsh preservation procedures such as freeze drying^28^ as well as drastically slower accumulation of biomass compared to other hosts such as yeast. Specifically, the inability to maintain cold-chain requirements, necessitates small-scale, portable drug production platforms to facilitate wider access to needed medications. As such, many groups have turned to the methylotrophic yeast *Pichia pastoris* (*Komagataella phaffii*) for applications in outside-the-lab scenarios as this host requires simpler media and shorter processing times for recombinant protein production^29^, as well as their tolerance to freeze drying^30^. *P. pastoris* is a preferred host for the production of complex recombinant proteins over other simple hosts, such as *E. coli and S. cerevisiae*, given its amenability to the production of compounds, such as protein therapeutics, with mammalian glycosylation profiles^31^. Furthermore, for rapid deployment, large-scale fermentation is not desired. To this end, Perez-Pinera *et al*. have engineered *P. pastoris* with inducible and switchable production of two distinct biologics (rHGH and IFNα2b) in a manner that is well-suited for a milliliter-scale, table-top microfluidic reactor capable of production of single-dose levels of product within 24 h^32^. This unprecedented production of two distinct biopharmaceuticals highlights a strategy for increasing production speed through the ability to use the same biomass to produce multiple products, as well as serves as a demonstration of decreased reactor footprint needed to produce complex proteins. Implementation of additional orthogonal genetic circuits in platforms like this could enable multiplexed production of a large array of therapeutic compounds, which could be switchable based on the presence of distinct stimuli (even leading toward a combined diagnosis and therapeutic production unit). This enhanced degree of production flexibility could be especially useful in applications such as space missions or active military missions, where oscillating demand for different therapeutics may occur in conjunction with limited access to space for carrying production units dedicated to single products. To address some of the field-deployable limitations of on-demand platforms (including expression limitations and downstream separations), Crowell *et al*. have developed the InSCyT (Integrated Scalable Cyto-Technology) platform for automated, cell-based, table-top, multiproduct biomanufacturing capable of end-to-end production of 100–1000 s of doses in around 3 days depending on the therapeutic compound^33^. This platform enables in-line, automated modules for *P. pastoris*-based production, purification, and final formulation steps, to yield clinical quality recombinant protein therapeutics in point-of-care settings. Furthermore, the use of continuous perfusion fermentation facilitated a decrease in bioreactor footprint size (the system utilizes a sub-liter bioreactor compared to the industrial 1000+ liter scales from which biologics are typically produced), enabling the entire platform to fit on a benchtop. While the need for electricity and pure oxygen inputs to run the system prevents its use in truly off-the-grid settings, the platform’s relatively simple technological requirements (especially given that its peristaltic pumped-based liquid handling could be amenable to recently reported low-cost, open source, 3D-printed peristaltic pumps^34^) could enable its deployment in resource-limited settings such as active military and space missions.

### Biotic/abiotic interfacing

Beyond the development of distinct whole-cell production platforms, several groups have demonstrated the potential for interfacing synthetic biological systems with abiotic systems to facilitate deployment in outside-the-lab settings. An example of particular relevance for enhancing platform portability and stability in diverse climates, as well as potentially enabling production capacity in diverse cell types, involves the coupling of materials science and engineered biology to yield encapsulation-based production platforms. For instance, Gonzalez et al. encapsulated *Bacillus subtilis* spores, known for resilience to many extreme stresses^35,36^, within 3D-printed agarose hydrogels for on-demand, inducible production of small-molecule antibiotics^37^. The customizable materials were demonstrated to maintain cell viability following applied stresses of ethanol, UV light, radiation, high osmolarity, and low pH. Johnston et al.^38^ recently developed an on-demand production platform that utilized functionalized pluronic hydrogels, featuring temperature-responsive and shear-thinning properties to enable homogenous cell distribution and extrudability, respectively. The mechanical robustness of these gels provided protection from cryopreservation stress for both bacteria and yeast cells. Furthermore, the platform versatility was demonstrated through both continuous and on-demand production of a wide range of value-added compounds from both mono- and cocultures, including L-DOPA, 2,3-butanediol, ethanol, colicin V, and betaxanthins. The ability to physically separate cocultures within separate hydrogels enabled facile manipulation of consortium population dynamics, minimizing needs for genetically encoded mutualism. Building off of this technique, Yuan et al.^39^ recently demonstrated portable, reusable, on-demand production of recombinant proteins up to 150 kDa from encapsulated *P. pastoris* cells via the Bioproduced Proteins On Demand (Bio-POD) platform. Importantly, these cell-laden material platforms are resilient to lyophilization, thus allowing for ambient storage and simple on-demand production. This feature is especially important for the case of recombinant protein production, typically marked by rigorous cold-chain requirements. Also, the capacity for encapsulation to enhance organism stability could be translated to production systems involving nonconventional hosts to expand the range of cell types applicable for production in outside-the-lab settings. Across the board, a major challenge still remains in the interfacing of these synthetic biology platforms with proper downstream purification modules, although some progress has been made in automated purification module systems^33,40^.

As highlighted earlier, on-demand and continuous bioproduction requires the preservation of metabolic activity in diverse settings. The use of microbially induced calcium carbonate (CaCO_3_) precipitation (MICP) to yield functional, regenerable bio-cements could provide an avenue for efficient, stimuli-responsive production of structural materials in outside-the-lab settings. One recent study engineered ureolytic *E. coli* to control biogenic CaCO_3_ properties by genetically manipulating precipitation rates thus demonstrating the potential to yield advanced functional materials with genetically modified organisms^41^. To enhance living building materials (LBMs) for harsh environments, another recent study developed LBMs composed of sand-gelatin hydrogel scaffolds containing cyanobacteria (*Synechococcus*) capable of MICP^42^. The resulting materials were regenerable for at least three successive rounds via the use of temperature and humidity switches, with higher microbial viability within the LBMs after 30 days than previously reported data (when maintained with at least 50% relative humidity). In addition, the materials could be recycled to serve as the abiotic component of new LBMs once no longer viable. However, the LBMs developed so far exhibit a clear tradeoff between maximizing cell viability (achieved at high relative humidity) and maximizing mechanical performance (achieved at maximum dehydration), requiring further work to improve the stress tolerance of the encapsulated organisms.

### Cell-free production platforms

Cell-free production platforms provide a viable alternative to traditional cell-based platforms for applications in protein and small-molecule production^18^. They are typically more readily amenable to the preservation, with two-month shelf stability demonstrated via lyophilization of *E. coli* crude extracts^43^, as well as three-month storage at elevated temperature (37 °C) achieved via the addition of cryoprotectants and separate storage of reaction components prior to preservation^44^.

In addition to enhanced storage capacity, cell-free production platforms have been adapted for use in remote, low-resource settings. As examples, Pardee et al.^45^ developed a freeze-dried, paper-based platform for on-demand biomolecule manufacturing in which freeze-dried cell-free systems and DNA constructs can be rehydrated to yield functional products via multi-enzyme pathways in a combinatorial manner. By combining cell-free protein expression with in-line purification capabilities, Sullivan et al.^46^ developed a rapid process for producing µg to mg quantities of therapeutic proteins including recombinant human erythropoietin (rhEPO) and recombinant human granulocyte-macrophage colony-stimulating factor (rhGM-CSF) using *S. cerevisiae* and *E. coli* cell-free expression systems respectively. Integrating cell-free expression with microfluidics, Murphy et al.^47^ developed the “Therapeutics-On-a-Chip (TOC)” platform for point-of-care cell-free production of therapeutic proteins. This platform integrated continuous-flow production and batch purification to produce a dose of the antimicrobial peptide within 6 h. By further incorporating automation, Adiga et al.^48^ developed an automated, portable Bio-MOD system utilizing lyophilized Chinese hamster ovary cell extracts to yield an end-to-end cGMP-quality manufacturing process in under 9 h. Important for outside-the-lab applications, both this platform and the TOC can fit in a briefcase to facilitate portability. The platform developed by Adiga et al. is equipped with self-monitoring process analytical technology (PAT) software for on-line absorbance, pressure, and temperature measurements, with further automated analytical technologies currently in development that enable the system to run through an entire process with minimal input from the system operator to enable use by non-experts.

### Ongoing challenges and needs

Many challenges remain in terms of deploying these platforms in the field. Generally speaking, whole-cell platforms can readily enable large-scale production via accumulation of biomass and have been demonstrated to be more amenable to adding post-transcriptional modifications to protein products compared to cell-free platforms. Thus, these platforms are best suited for applications in which large quantities of product are needed, as well as for the production of compounds with complex glycosylation profiles. Whole-cell platforms could benefit from efforts to enhance organism resilience to metabolic and environmental stresses. In terms of environmental stress resilience to afford shelf stability and long-term performance (a trait required for continuous production platforms in variable climates), further developments in strain engineering can be coupled with ongoing developments in biotic/abiotic interfacing to yield more robust platforms. In addition, the design of synthetic circuits resistant to mutation so as to enhance genetic stability of organisms would enhance platform hardiness, a major concern for platforms that need to operate under harsh environmental conditions and/or long time periods in which cells undergo high levels of mutational stress to retain viability. The development of polymers and fabrication methods compatible with a more diverse range of cell types could help expand the range of host organisms applicable for outside-the-lab production schemes beyond those known to be more stress-resilient (yeast and bacteria). Conversely, the propensity of certain organisms (such as *B. subtilis* spores) to withstand extreme stressors highlights a need to focus additional strain engineering efforts on production from additional hosts known to be stress resilient to help bypass limitations in deploying living cells in variable climates. Thus, engineering efforts in materials science as well as biology could help make outside-the-lab deployment of whole-cell production platforms a reality. Conversely, cell-free platforms are more easily amenable to being made shelf stable and can yield faster production times due to the elimination of the biomass accumulation step (with a clear tradeoff in the amount of product made per reaction). Thus, these platforms are best suited for situations in which small quantities of product are needed quickly (i.e. 4-6 h instead of several days). Progress in the design of reaction systems to yield larger production titers is needed to enable the use of these systems for production applications that require more than a few doses at a time. Developments in cell-free protein synthesis to enable the production of proteins with complex glycosylation patterns will be required to expand the range of achievable products.

Both whole-cell and cell-free systems would benefit from advancements in platform automation to truly enable autonomous performance. While many of the systems discussed have incorporated automation elements, such as real-time process monitoring of production and purification status, further improvements to enable end-to-end production of products formulated for consumption with minimal user inputs required (ideally the push of a ‘start’ button) would help minimize the need for professionals to run the equipment in the field. In addition, efforts to bring down system costs will likely be required to make on-demand production platforms deployable in low-resource settings. Some strategies to achieve this include (1) focusing on production from low-cost organisms such as bacteria and yeast, (2) consolidated, continuous production and purification within singular platforms, (3) engineering strains or cell-free reaction mixtures capable of multiplexed production of distinct products either simultaneously or with production ratios controlled by inducible expression, as well as (4) lowering costs of production and purification machinery possibly via 3D printing devices from low-cost starting materials. While some of these strategies are employed separately in the platforms discussed above, combining them within each platform will likely be necessary to enable economic viability.

One potential strategy to expand use to extreme settings with limited or no power access could be to couple platforms with microbial fuel cells, whereby bio-catalytic functions of living cells can be used to convert chemical to electrical energy to power end-to-end production. While further advancements in aspects such as increasing electron transfer rate, decreasing biofouling and catalyst deactivation, and limiting excessive biofilm growth are required to improve currently achieved efficiencies^49^, ongoing advancement in this area could help expand the range of outside-the-lab settings that on-demand production platforms could serve. In addition, the need for pure oxygen inputs limits the applicability of currently developed whole-cell platforms. Elimination of these requirements will likely require either metabolic rewiring of commonly used bioproduction organisms to lower oxygen demands (and enable operation from air rather than pure oxygen) or engineering bioproduction capabilities in organisms that contain metabolic architecture for survival in oxygen-starved environments (such as *P. aeruginosa*^50^). Finally, increased regulatory approvals will be required to deploy these on-demand production platforms in the field. Given that there are already FDA-approved products from continuous whole-cell production platforms, the approval process for these platforms will likely primarily require demonstration of sufficient product safety and efficacy as those made from approved processes. As there are currently no FDA-approved therapeutics produced via cell-free systems, these platforms will likely face a more arduous path to approval. However, Adiga et al.^48^ did incorporate guidance from the FDA Emerging Technology Team on steps needed to enable regulatory acceptance of their products, and Sutro Biopharma is currently undergoing the FDA approval process for therapeutics produced via cell-free manufacturing^51^. Once a cell-free production platform is on the market, approval of cell-free production platforms will hopefully be as (relatively) readily achievable as that of whole-cell systems.

### Future applications for on-demand outside-the-lab production platforms

Platforms such as those described above could be used in remote military missions or space missions wherein possible medicines or supplements can be manufactured quickly in the event of a sudden disease outbreak. Similarly, on-demand production platforms could greatly benefit communities in developing nations, which often lack the infrastructure to store or access treatments. In both of these circumstances, portable, modular production platforms could be stored at room temperature and rehydrated/activated when a need for a certain compound arises, ideally to then yield fully purified products within a few days. In addition to therapeutic production, platforms for on-demand production of speciality chemicals can be used to minimize the need for inventory stockpiling in settings with limited storage capacity or infrastructure, enabling rapid production ramp-ups in response to oscillating market demands. Of particular interest for military missions, on-demand biofuel production platforms could obviate the need to transport and store large quantities of fuel on long missions. Platforms for continuous or on-demand production of small molecules can also be useful in terraforming operations as well as bioremediation. The development of living building materials, especially those that can develop and function using simple inputs such as sunlight, could facilitate the development of structural infrastructure in remote areas. In particular, these platforms could enable the production of buildings in outer space, in which transport of heavy building materials from earth is not feasible. The concept of living building materials can be extended further to the concepts of in situ production of protective coatings and anti-corrosion agents onto buildings, ships, and other parts of the built environment. Intentional coatings/doping of surfaces with microbes can be useful for in situ production of antimicrobial peptides, hydrophobic agents, or even anti-freeze proteins. These varied applications can help enhance material performance. Overall, ongoing development in whole-cell, cell-free, and biotic/abiotic interfacing production systems can have wide-ranging impacts on diverse outside-the-lab scenarios.

## Biosensing

Biosensing has broad applications including disease diagnostics, hazard detection, food/water security, and actuator functions and can utilize a variety of sensing modalities from nucleic acids to whole-cells^52^. While a range of biosensor functions have been catalogued elsewhere^13,53–60^, less attention has been dedicated to the development of biosensing platforms for use outside-the-lab. In such applications, synthetic biosensors (from biomolecules to whole-cells) can be used in low-cost, rapid diagnostics, as a continuous monitoring system for health-related biomarkers, as a living dosimeter to detect for exposure hazards, or even as a deployable and dispersible sensor whose signal can be detected from a distance. In most of these applications, the integration of biology with device fabrication (whether purely a physical material or a larger microfluidic/electronic interface) enables the quick transduction of biosensing to signal, as well as autonomous functioning (Fig. 2).

### Cell-based biosensing platforms

Biosensing requires cells to maintain a sustained surveillance mode followed by a rapid response. In this regard, the use of spores and biofilms has been explored to detect external signals and elicit synthetic programmed responses. Some biofilm-based developments have used amyloid fibers, produced by the bacteria, as scaffolds to yield artificial biofilms and diverse extracellular matrices^61–63^. In these applications, embedded bacteria can be engineered with synthetic gene circuits to self-organize into desired patterns (i.e., without pre-patterning or addition of morphogen gradients) in order to maximize material tunability^61,64^. Recently, Huang *et al*. applied this approach to develop programmable, secreted TasA fusion proteins from *B. subtilis* to yield self-assembled biofilm scaffolds with tunable properties that also maintained viability for at least five weeks without nutrient supplementation^65^. In a biosensing proof-of-concept application, the engineered biofilms could be programmed to elicit fluorescent output in response to inducers (in this case, IPTG).

In addition to whole-cells, some groups have considered the use of bacteriophages for biosensing platforms, given their high recognition specificity for their target bacterial hosts such as *E. coli*^66^ and stability under thermal and chemical stresses^67,68^. Several platforms have utilized immobilization of phages isolated from sewage for use in the specific, sensitive detection of pathogenic bacteria^69,70^, with a recent application coupling engineered M13 phage with gold-binding peptides and gold nanowires to yield an aggregation-free colorimetric sensor for heavy-metal ion detection^71^.

A set of promising technologies to enable rapid, outside-the-lab application includes the integration of sensing systems with deployment technologies such as microfluidic devices and paper-based platforms, ideally with automation capabilities. Some recent developments in this area include the development of a microchemostat device for portable, parallel culture chambers for rapid, sensitive, and simultaneous heavy-metal ion detection^72^, a portable, high-throughput microfluidic bioluminescent whole-cell sensor array on a chip, with integrated sampling, incubation, and photodetection modules^73,74^, and a fully automated biosensor device for mercury detection in water^75^ comprised of *Pseudomonas putida* and *Aliivibrio fischero* for testing of freshwater and saltwater samples, respectively. Furthermore, the coupling of machine learning with high-throughput microfluidics has recently been demonstrated to enable continuous, simultaneous tracking of transcriptional network dynamics of thousands of *E. coli* strains at the genome scale^76^. Real-time detection of the heavy metals in field samples has been demonstrated as a proof-of-concept application of such a platform for advanced biosensing. Some groups have directed attention to developing paper-based, whole-cell biosensing platforms given their low-cost, portability, and facile manufacturing. An example application includes the detection of pathogenic bacterial contamination (via detection of quorum-sensing molecules) in food samples using microdots of immobilized *E. coli*-based sensors on paper^77^.

### Biotic/abiotic interfacing

As with bioproduction, several groups have demonstrated the potential for interfacing synthetic biological systems with abiotic systems. In particular, the preservation of cells (especially through 3D printing and embedding in materials) can enable the sustained cellular viability required for long-term outside-the-lab sensing. The regenerable bacterial biofilm platforms described above are an excellent example of engineering cells to both carry out biosensing functions and produce their own encapsulation material for enhanced stability. The encapsulated spore-based platform developed by Gonzalez et al.^37^ the combined use of a stress-resistant organism with a durable, portable and easily customizable material scaffold to yield a highly resilient platform for simple genetically encoded small molecule *B. subtilis* spore sensors even following extreme stresses, highlighting its potential to house more complex spore-based sensing systems in harsh environmental conditions. Another approach for cell-encapsulation was employed by Liu et al.^78^, in which stretchable, strong, biocompatible hydrogel-elastomer hybrid materials were used to encapsulate engineered bacteria. In this application, cells retained a sense and response function across 3 days without the addition of media. Moreover, this material was mechanically resilient and free from cell leakage under applied deformations of stretching and twisting, thus yielding a stretchable, flexible material enabling attachment to skin for wearable sensing applications. The implementation of the stress-protective encapsulation techniques detailed so far could facilitate the commercial realization of wearable biosensors^79^.

Given the resilience of spores to a multitude of stresses, many groups have explored 3D ‘cell-in-shell’ structures also known as ‘artificial spores’, formed by encapsulating individual cells within an artificial shell to yield a mechanical stable, selectively permeable, and chemically functionalizable system that can preserve stress-susceptible organisms including mammalian cells and Gram-negative bacteria^80^. Recent efforts have focused on developing artificial spores that can both provide robust stress protection and can also degrade under cytocompatible conditions, a necessary characteristic to enable function of the cell-based sensor after degradation of the shell.

Some major strategies to achieve this include coatings based on metal-ligand coordination complexes^81^, degradable polymer nanoshells^82^, and self-repairing nanoshells^83^. Nevertheless, further work is needed to fully integrate artificial spores into functional, portable biosensing platforms.

In addition to these proof-of-concept systems, encapsulation-based platforms can be deployed on a much larger scale for biosensing, such as alginate-encapsulated bacterial biosensors engineered to detect TNT with quantification via a laser-based optoelectronic remote detection system^84^. The system served as a proof of concept for safe, remote detection of buried land mines, with further improvements needed including strategies for removal of biosensors after use, decreased response time, and improved stability under variable climates.

The combination of encapsulation techniques along with fluidics systems has enabled portable and easily deployable biosensing systems that often bypass cold chain requirements. As an example, a portable system for spore-based whole-cell biosensing of arsenite and zinc in a centrifugal compact disk microfluidic platform was functionally stable for 1 year under elevated temperature and a variety of humidity/drought conditions^85^. A living material polymer device containing *B. subtilis* spores encapsulated in a PVA matrix for continuous sensing of small-molecule analytes could be stored for extended time periods at room temperature without compromising sensor function^86^. Incorporating hydrogel encapsulation with a paper-based device, Weaver et al.^87^ first developed a whole-cell yeast paper-based biosensor device to yield a biologically based paper analytical device (BioPAD), which could be stored at 4 °C and 37 °C and remain viable for at least 6 months and 56 days respectively. As a demonstration of a user-friendly culturing device, Huacca et al.^88^ developed a portable platform for bacteriophage or bacteria amplification made from pre-patterned paper, tape, and a PDMS membrane that was able to be constructed by high school students in Nairobi, Kenya, which could be integrated with biosensing platforms.

Interfacing cells with microelectronics has widespread applications in efficient sensing and actuation systems such as diagnostics, high-throughput drug screening, and hazard detection^89^. The seamless integration of biology with microelectronics can provide unique applications including hybrid biosemiconductor platforms containing self-powered, on-chip Intelligent Sensing Systems (ISS) composed of living cells for carrying out power generation and sensing. This coupling could also be used to generate complementary metal oxide semiconductor (CMOS) technology for information storage and computation functions. One recent advance in bio-semiconductor platform development includes an ingestible micro-bio-electronic device (IMBED) utilizing engineered probiotic *E. coli* NISSLE 1917 integrated with semiconductor microelectronics for in situ gastrointestinal biomarker detection via a bioluminescent detection circuit that communicates with an external device^90^. An additional example is the development of a quantitative, portable CMOS lab-on-chip platform coupling nucleic acid amplification-based sensing with ion-sensitive field-effect transistors (ISFETs) for efficient chemical-to-electronic detection of the most common worldwide malaria-causing parasite *Plasmodium falciparum*. Furthermore, the system has the added capacity to differentiate between parasites resistant to artemisinin^91^, thus enabling more specific treatment of this disease. Beyond hybrid semiconductor-based platforms, another recent advance involved interfacing cells engineered with a population control genetic circuit with microelectronics for efficient sensing of the circuit output. The platform bypassed the need for an optical output by using cell lysis-mediated impedance measurements^92^. Such an approach has widespread applications such as in heavy-metal sensing, oscillatory synthetic circuits, and hybrid computational device generation.

### Cell-free biosensing platforms

Cell-free biosensing can provide an alternative to cell-based platforms to yield sensitive, specific detection without the limitations of handling and maintaining the viability of whole-cell systems. In particular, crude extracts used to carry out cell-free gene expression (CFE) can activate complex gene expression cascades and metabolic pathways. A recent field example is the use of a cell-free biosensor to detect the environmental toxin cyanuric acid (CYA) in water through coupling *E. coli* gene expression machinery with a non-native transcriptional regulator from *Pseudomonas* sp., via engineering of hybrid *Pseudomonas-E. coli* promoters capable of functioning in a freeze-dried *E. coli* cell-free system^93^. To expand the collection of detectable analytes beyond nucleic acids and a specific set of well-characterized chemical contaminants, Silverman et al.^94^ developed a platform for organic molecule detection via coupling metabolic conversion of target molecules with transcription-factor-based biosensing for efficient detection of atrazine. This modular platform enabled multiplexed sensing all within one mixture via the addition of multiple cell-free extracts with specific reporter plasmids. Both systems could be freeze-dried until needed, rehydrated with a water sample of interest, and detectable via fluorescent signals observable within an hour. In a similar vein, Voyvodic et al.^95^ demonstrated the use of modular cell-free sensors coupling metabolic cascades with transcription-factor-based biosensors. The platform was shown to function in complex solutions, such as commercial beverages and human urine samples, requiring only dilutions of the original samples to function properly. Working towards the development of reliable, point-of-care biomarker quantification, McNerney et al.^96^ developed a platform using a parallel calibration scheme in which patients’ own blood samples are used to generate a calibration curve through the use of both transcription factor- and toehold switch-based sensors in an equipment-free manner. With the goal of enhancing user-friendliness beyond tube-based reactions, Gräwe et al. developed a paper-based, cell-free sensing platform capable of detecting heavy-metal ions in water that paired to allosteric transcription factors within the cell-free protein synthesis system^97^. In doing so, the freeze-dried reagents on paper could be rehydrated with a sample of interest and the signal can be quantified using a smartphone camera combined with a dual light filter system.

Many cell-free platforms have leveraged recent synthetic biology advancements of CRISPR/Cas-based systems as well as programmable RNA switches. For instance, Pardee et al.^98^ utilized toehold switches to develop orthogonal, cell-free genetic circuits capable of discerning between mRNA from different Ebola strains using paper-based freeze-dried cell-free extracts stable at room temperature for over one year. Later, this same group built upon these nucleic acid-based diagnostics sensors to develop a rapid platform for sequence-specific Zika virus detection using an isothermal RNA amplification and CRISPR/Cas9-based analysis technique^99^. A paper-based, cell-free sensor platform coupling toehold switches and nucleic acid sequence-based amplification (NASBA) was also developed to analyze microbiome samples that could detect mRNA from 10 different species of disease-relevant bacteria in a manner on-par with RT-qPCR without the need for such expensive equipment^100^. To further enhance the multiplexed reporting of gene-circuit-based sensors, Mousavi et al.^101^ recently developed a gene circuit/electrochemical interface for efficient reporting from restriction endonucleases via DNA-functionalized nanostructured microelectrodes with widespread applications such as parallel detection of up to 10 antibiotic resistance genes.

Harnessing the ssRNA *trans*- cleavage activity of CRISPR effector Cas13, Gootenberg et al.^102^ developed the Cas13a-based nucleic acid detection platform, Specific High-Sensitivity Enzymatic Reporter UnLOCKing (SHERLOCK), capable of detecting viruses and pathogenic bacteria at attomolar levels. All SHERLOCK components can be combined in one reaction, with high sensitivity and stability, maintained upon freeze-drying onto paper, and subsequently rehydrated upon demand. Highlighting the potential for multiplexed CRISPR strategies to facilitate multi-input biosensing, the group updated this system to develop SHERLOCKv2 which utilized orthogonal Cas13 and Cas12 enzymes as well as the combination of Cas13 with CRISPR type III effector nuclease Csm6 to increase sensitivity, and implemented a lateral flow readout to enhance user-friendliness for point-of-care setttings^103^. Further development included pairing the heating unextracted diagnostic samples to obliterate nucleases (HUDSON) protocol with SHERLOCK to enable instrument-free diagnostics directly from patient samples^104^.

Other groups have developed portable, field-ready biosensing platforms based upon CRISPR-Cas12 effectors that carry out both *cis-* and *trans-* cleavage activities on ssDNA^105^. These include the one-hour low-cost multipurpose highly efficient system (HOLMES) which couples the DNA targeting and collateral cleavage activities of Cas12a with a quenched ssDNA reporter to yield efficient attomolar detection of target DNA^106^ and the DNA-endonuclease-targeted CRISPR trans reporter (DETECTR) that uses a one-pot nucleic acid-sensing method^107^. Both methods enable sensitive detection of DNA without the need for prior in vitro transcription to RNA, as required by the Cas13-based platforms. A particularly relevant application includes using DETECTR in a lateral flow assay (LFA) to identify SARS-CoV-2 from respiratory swab RNA extracts within 40 min^108^. Ding et al.^109^ have also recently demonstrated a one-pot CRISPR-Cas12a-based assay (involving the coupling of two CRISPR RNAs without limitation of protospacer adjacent motifs) capable of detecting as low as a few copies of the virus within 20 min. Furthermore, this platform can produce a signal visual by eye with the aid of an LED light using a store-bought hand warmer as the incubator.

Several groups have demonstrated proof-of-concept field deployment of other kinds of cell-free sensing platforms, a portable colorimetric detection platform for sensing of endocrine-disrupting chemicals using cell-free protein synthesis of an allosterically activated fusion protein^110^, as well as a riboswitch-based sensor for colorimetric detection of fluoride contamination in groundwater samples which was capable of detecting concentrations around 2 ppm (the EPA limit)^111^. Jung et al.^112^ demonstrated detection of a wide range of common water contaminants in municipal water samples including small molecules, metals, and antibiotics using RNA Output Sensors Activated by Ligand Induction (ROSALIND), a sensing platform employing allosteric regulation of in vitro transcription of fluorescence-activated RNA aptamers. While fluorescence outputs are more difficult to detect by the naked eye than colorimetric, Jung et al. further demonstrated coupling of the platform with a 3D-printed handheld device which improved visualization of the signal. All three of these platforms were demonstrated to be lyophilizable and shelf stable for at least 2.5 months. In addition, they all require 37 °C incubation which has been demonstrated to be achievable using a low-cost handwarmer as well as by using a patient’s own body temperature^111^. Overall, the field-ready features demonstrated by these platforms are quite promising in terms of enabling their deployment in settings with limited or no power.

### Ongoing challenges and needs

As evident in the wide range of platforms discussed, biosensing is arguably the most developed application space discussed in this Perspective with respect to recent innovation for deployment in outside-the-lab settings. Collectively, the coupling of further advancements in synthetic biology along with shelf-stable, user-friendly platforms has the potential to generate a vast array of highly sensitive and specific outside-the-lab sensing platforms. An advantage of whole-cell platforms includes the wider range of currently developed detection capabilities, compared to those of cell-free detection systems (which have been largely limited to nucleic acid detection before the recent developments in metabolic transducer modules discussed above^95^). Furthermore, whole-cell systems are better suited for long-term continuous sensing applications beyond simple analyte detection within a sample (such as continuous hazard detection on military missions as well as wearable sensors for long-term health monitoring) given their ability to regenerate over time. However, several challenges prevent the ease of deployment of such systems in variable outside-the-lab environments. Whole-cell biosensing platforms would benefit from enhanced storage stability and stability while in a metabolically active state if deployed for continuous sensing. This will likely require both additional strain engineering efforts as well as the development of enhanced polymer encapsulation strategies via encapsulation in chemically derived polymers or biologically derived polymers such as biofilms. Directing more effort to the development of biosensing capabilities in naturally stress-resilient systems, such as phages and spores, could serve as an additional avenue to achieve sensitive, specific systems with sufficient stress-resistance for deployment in variable environmental conditions. In addition, combining more of the systems utilizing encapsulation and cell-in-shell strategies with user-friendly interfaces will be vital to enabling their deployment. This coupling may uncover additional challenges associated with interfacing platforms based upon different polymer stabilization techniques and will not necessarily be a trivial task. Furthermore, the design of synthetic circuits resilient to mutation to enhance the genetic stability of organisms would enhance platform robustness, especially for long-term continuous sensing applications.

In the case of cell-free platforms, deployment in outside-the-lab scenarios can be achieved more readily, with several examples of field-deployed platforms for diagnostics and water quality monitoring. While shelf stability concerns are less of an issue for these platforms (although still nontrivial as shelf stability beyond 2–3 months has yet to be demonstrated for many systems), a major limitation of cell-free detection is the more limited range of detectable compounds with respect to whole-cell platforms. Some cell-free platforms discussed earlier demonstrate feasibility for expansion of detectable compounds via the use of transcription factor-based sensing as well as coupling metabolic transformations to convert an analyte into a compound detectable by a developed sensor. Further advancements in these areas, such as expanding the range of allosteric transcription factors beyond those that function by preventing RNA polymerase extension unless bound to the cognate ligand, as well as additional work to characterize and employ more complex metabolic transformations to convert additional targets to compound’s detectable by developed sensors. An additional limitation of cell-free sensing platforms includes difficulty in large-scale purification of reaction mixture components. Efforts to develop methods for consistent purification of reaction components, such as transcription factors, at a large and cost-effective scale will be required for large-scale deployment. In addition, further work is needed to develop many cell-free technologies into fully integrated platforms with enhanced multiplexing, increased speed, and improved portability (potentially through embedding in polymers or freeze-drying on paper). Moving towards this goal, Hajian et al. have developed the CRISPR-chip platform utilizing immobilized dCas9 coupled with single-guide RNA to yield sensitive, fast electrical detection of target nucleic acids bypassing the need for amplification. For the case of platforms that require multiple sampling processing steps, further work is necessary to integrate with additional user-friendly platforms such as LFAs, microfluidic paper-based analytical devices (µPADs)^113^, and polymer microfluidics. Ideally these systems could be further incorporated with smartphone analysis capabilities to enable low-cost, efficient diagnostics for health and hazard detection in a similar fashion to other reported biosensing devices^114–118^.

Beyond advancements specific to whole-cell and cell-free systems, both platform types would benefit from increasingly sophisticated genetic circuits. The development of increasingly complex circuits could have broad impacts on biosensor design including the potential to expand the range of detectable analytes through the development of new sensing control mechanisms, as well as the limitation of crosstalk in multiplexed reaction environments via stricter regulation over ligand specificity. While many platforms discussed in this section center around chemical inputs, the development of sensors responsive to non-chemical stimuli could expand the scope of applications of outside-the-lab biosensing. For instance, the development of biosensors responsive to gravity (possibly through the engineering of organisms known to elicit responses to changes in gravitational forces such as *C. elegans*^119^) could enable field-deployable gravimeters used to detect mineral deposits in remote regions or even to analyze the structure of planets on space missions. Many of the platforms discussed above-required sample processing prior to sensing. Implementation of more user-friendly interfacing, either via one-pot sensing reactions that permit input of unprocessed samples or through integration with microfluidics and/or paper-based interfaces to carry out sample processing steps without requiring user input beyond sample addition, could enhance platform usability in low-resource settings. This interfacing will likely require additional engineering of sensing circuits in some cases to enable the processing of complex mixtures.

### Future applications for on-demand outside-the-lab biosensing platforms

Biosensing platforms have the potential to transform the safety and efficiency of human performance health such as in remote military missions, space missions, or even in early health event monitoring for at-risk patients. For instance, biocompatible, wearable, or implantable hydrogel-based sensors can be utilized in the field (where there would be limited access to power sources as well as exposure to potentially harsh environmental conditions) to continuously monitor soldier health. Beyond monitoring vital signs, the real-time detection of biomarkers using cellular detectors can proactively monitor potential health and safety issues for individual soldiers on training missions. Likewise, biosensing platforms could also be utilized for biometric applications and identity verification^120^, serving as a barrier to security threats in areas where limited security infrastructure exists. Continuous health sensors and diagnostics tests can seamlessly integrate with the WHO’s initiatives to promote widespread adoption of mHealth (mobile health)^121^ to enable individuals in remote regions to interact with healthcare professionals to obtain timely care. Wearable or implantable biosensing devices that could transfer data via Bluetooth could be utilized to alert healthcare professionals if an individual is consistently showing symptoms that warrant medical attention, without requiring in-person visits which may not be possible for individuals in remote regions. Fully integrated, field-deployable wearable platforms have not been widely demonstrated due to ongoing concerns^79^ related to issues such as long-term sensor stability concerns and the need for demonstration of sensing capabilities beyond a few simple biomarkers. Ideally, integration of encapsulation techniques discussed in this section together with more complex genetic circuits and improvements in sensor stability in metabolically active states could enable deployment of these platforms in outside-the-lab settings.

Likewise, portable, stable biosensors for hazard detection can serve as real-time in vivo dosimeters detecting potential threats encountered in the field on remote military missions or the hazards of space missions, in which sensors would have to function continuously over long time periods and remain viable in austere conditions. For hazard detection in the field, stable whole-cell or cell-free biosensors engineered for fluorescence or luminescence-based output could be sprayed onto large environmental areas and have the signals monitored either periodically through drone-based detectors^122^ or continuously via satellite-based luminescence sensors^123^. In non-transparent settings (such as the soil) which complicate the ability to carry out many common sensing operations, the use of alternative sensor outputs could expand the range of environments in which continuous detection is possible. For instance, recent work developing biosensors with gas reporters, which can be implemented in common sediment bacteria such as *S. oneidensis*^124^, highlights the potential of utilizing non-visual reporters to enable sensing in variable settings. Furthermore, continuous biosensors could be used for individual monitoring through integration with clothing or even as an airborne-biohazard system that could analyze individual breath samples.

The use of synthetic biosensors for hazard detection is also especially prevalent in developing nations for the detection of hazards and contaminants such as pathogens, chemical contaminants such as metals, agricultural products, and pharmaceuticals in drinking water^125^, and harmful bacteria in food^77^. Furthermore, real-time monitoring of agricultural land health can identify locations of excess pesticides and ripening agents to determine optimum dosages for future crops. In this regard, biosensors could be sprayed onto crop fields and analyzed for compositions of relevant analytes, such as residual pesticides or heavy metals, to promote enhanced food safety. As with the discussion on bioproduction above, the combination of these biosensing platforms with living materials and other parts of the built environment could ultimately create an “Internet of Living Things” through which biosensing plays a critical role in detecting and responding to hazards or other issues.

## Closed-loop living therapeutics and probiotic delivery

While therapeutic delivery is a well-documented field, the combination of biosensing and therapeutic delivery mechanisms through the use of synthetic biology can create hassle-free treatment options for patients with limited access to medical facilities. Such closed-loop therapy systems include a continuous sensor for an analyte or signal of interest, a control algorithm for determining needed therapeutic dosing based on the input signal, and an actuator to drive therapeutic delivery to the patient in response to the controller without the need for external intervention^126^. An area of particular interest that we discuss here is the coupling of synthetic biology advancements with closed-loop delivery to develop whole-cell solutions for both detection and subsequent treatment against bacterial infections. In a different, but related, context, the closed-loop delivery platforms are relevant for soil microbiome (rhizosphere) in situ pesticide and fertilizer production, which can also be accomplished via the delivery of engineered microbes. These outside-the-lab applications that take place on or within the human body, as well as in remote agricultural regions or in the soil, require new advances for synthetic biology. This section focuses solely on whole-cell and biotic/abiotic interfacing approaches, as cell-free approaches in this area have not been as heavily explored (Fig. 2).

### Whole-cell closed-loop therapeutics and probiotic delivery platforms

The ability to sense and detect/respond to pathogens has been a strong interest for synthetic biology^127^ with applications including wound healing and as preventive measures for bacterial infections. For instance, engineered microbes have been demonstrated to sense and respond to pathogenic infections inside the body, such as those caused by the opportunistic multidrug-resistant pathogen *Pseudomonas aeruginosa* (a particular concern in developing nations^128^). The use of quorum-sensing blocking approaches such as quenching behavior via engineered probiotic organisms, has the potential to combat chronic infection and antimicrobial resistance concerns^129^. For instance, the probiotic *E. coli* Nissle 1917 has been engineered to sense AHL (a secreted *P. aeruginosa* autoinducer) to induce its own lysin E7-mediated lysis which releases anti-*P. aeruginosa* toxin pyocin S5^127^. Further engineering of the strain to upregulate anti-biofilm enzyme dispersin B provided enhanced *P. aeruginosa* clearance in mouse models^130^. Notably, the most effective protection using this system was conferred in animal models that received a dose of probiotics 7 days before pathogen exposure, demonstrating that this strategy is most ideal as a preventive measure. Furthermore, to avoid the potential for horizontal gene transfer of an antibiotic-resistant marker to other bacteria in vivo, the work utilized an auxotrophic marker to promote biocontainment of the plasmids to the engineered strains.

Cholera, a life-threatening, often unreported gastrointestinal infection caused by *Vibrio cholerae* has been shown to primarily impact populations in developing nations in Sub-Saharan African lacking easy access to healthcare and sanitation^131^. The common probiotic bacterium *Lactococcus lactis* inhibits *V. cholerae* via its natural lactic acid secretion function and can yield a gut environment hostile to *V. cholerae* in mouse models^132^, indicating its potential as a therapeutic candidate. To enable deployment in a synthetic fashion, a synthetic gene circuit capable of sensing the cholera autoinducer 1 molecule was imported into *L. lactis* and detection was possible via secretion of β-lactamase into fecal output samples for point-of-use diagnostic applications. While the engineered *L. lactis* strains showed decrease protective effects against *V. cholerae*, likely due to increased metabolic burden from the synthetic circuit, a mixed population of engineered and natural *L. lactis* strains was sufficient to yield simultaneous protective and diagnostic effects.

Engineered cellular systems additionally have the potential to sense and treat non-pathogenic diseases such as diabetes and cancer. Many of these systems involve the use of engineered human cells to carry out sensing and responding to diseases via tightly regulated control strategies. For instance, Ye et al.^133^ developed a synthetic insulin-sensitive mammalian transcription circuit capable of sensing and reversing insulin resistance in vivo. Polymer encapsulation of the engineered cells and subsequent injection into mice was demonstrated to reverse insulin resistance for at least 20 days, demonstrating potential as a long-term treatment. Another recent example involved a closed-loop synthetic gene network in mammalian cells to sense liver disease-associated biomarkers and synthesize a protein therapeutic treatment in response^134^. Both of these systems utilized cell encapsulation within alginate-poly-(L-lysine)-alginate beads to promote long-term residence within the body (although further work to determine the lifetime of these devices in vivo is needed) and were implanted into the peritoneum of tested mice.

Beyond platforms aimed at delivery to the human microbiome, synthetic biology-enabled closed-loop delivery platforms are quickly being used to target the soil microbiome to help remote farm areas to both decreases nitrogenous fertilizer, which is both costly and poses environmental and health concerns^135,136^ as evident by the projected 12.8% growth in the biofertilizer market (currently a $1 billion market) from 2020 to 2027^137^, as well as reduce chemical pesticide application and improve plant health. Plant microbes have long been used to coat seeds to promote growth and reduce biotic/abiotic stresses. In this respect, just as the gut microbiome is a topic of strong recent interest, there is renewed interest in both understanding the interactions between plant roots and the rhizosphere^138,139^ as well as manipulating processes such as nitrogen fixation^140^.

In this vein, Fox et al.^141^ demonstrated the potential for in situ biological nitrogen fixation (BNF) by inoculating major cereal crops with the nitrogen-fixing rhizobacterium *Pseudomonas protegens* Pf-5 ×940, a strain developed to include a synthetic nitrogen fixation (*nif*) gene cluster from *Pseudomonas stutzeri* A 1501. While this approach increased crop biomass, there was a fitness burden on the bacterial cells resulting from constitutive nitrogenase activity. To address this issue, Ryu et al.^142^ engineered inducible *nif* clusters in the endophyte *A. caulinodans* to yield high levels of inducible nitrogenase activity without feedback ammonium repression. While further strain stability testing is needed, as well as allergenicity testing to determine whether the resulting crops yield additional allergies in humans, this work demonstrates the potential to link in situ production from synthetic organisms with crop production, especially aiding the crops yields for more remote or arid regions.

Broadly speaking, plant growth-promoting rhizobacteria (PGPR), which have a diverse range of functions including the propensity for nitrogen fixation detailed in the preceding paragraph, show potential as an environmentally friendly alternative to chemical pesticides and fertilizers^143,144^. In terms of developing alternatives to chemical pesticides, phytopathogens are known to utilize quorum-sensing to regulate the expression of pathogenic phenotypes, thus making quorum quenching strategies (i.e., degrading signaling molecules to reduce virulence without impacting pathogen growth) a promising strategy for promoting crop health^145^. Using such an approach, Rodriguez et al.^146^ demonstrated the ability of a *Pseudomonas segetic* strain P6 (isolated from the *Salicornia europaea* rhizosphere), to carry out both plant growth-promoting and QS-signaling molecule degrading functions, highlighting its potential as a deployable biocontrol agent. Similar to nitrogen fixation, allergenic and/or toxicity effects will need to be tested before being employed in full-scale agricultural settings.

### Biotic/abiotic interfacing

In the case of treatment of infections within the body, orally ingestible cell-based platforms are quite promising for deployment in outside-the-lab scenarios as the treatments are both user-friendly and require minimal equipment by virtue of their autonomous function within the gut or on the skin surface. One major requirement for outside-the-lab application involves establishing the long-term viability of these living systems which can include fluidized bed drying^147^, air-drying^148^, and lyophilization^149^, but further work is needed to improve these platforms for regions with varying temperatures. One alternative method to increase long-term storage capacity is the use of encapsulation that has been demonstrated to improve the long-term storage capacity of *E. coli*^38^. As these therapies and delivery vehicles are being ingested, biocompatibility tests of each system would be required. Nevertheless, the wide use of common encapsulation materials such as alginate and pluronic hydrogels in biomedical approaches^150^ suggests this approach could be a viable method to yield shelf-stable, effective prophylactic therapies for combatting bacterial infections. In the case of implantable devices or stents, it is important to mitigate the leakage of these organisms from within the material matrix out into the body. In this regard, the use of commensal organisms that are engineered via synthetic biology are important to mitigate any immunological response to released organisms.

In addition to the potential for sensing and fighting pathogens within the body discussed above, the interfacing of synthetic biology developments with materials science has applications in external wound healing. For instance, the resilient materials created from 3D-printed spores^37^ can detect quorum signals from *S. aureus*, a bacteria which commonly infects wounds, and produce GFP in response. Furthermore, *B. subtilis* strains capable of secreting lyostaphin and thiocillin, antibiotics effective against *S. aureus* infections, in response to IPTG or vanillic acid addition opens the possibility of creating a closed-loop approach, in which the engineered sensing and killing cells could modulate each other’s responses for point-of-care applications in wound healing. The cell-encapsulated, stretchable hydrogels^78^ also described above provide another promising mode of delivery for wound-healing treatments that could serve as a delivery vehicle for strains engineered to sense and respond to pathogenic presence on wounds.

Bio-polymer interfacing can also yield enhancement of PGPR delivery to crops. With the goal of enhancing organism stability and ease of PGPR delivery by farmers, Hussain et al.^151^ developed electrospun biocomposite polymer nanofibers for seed coating containing a *B. subtilis* and *Seratia marcescens* consortium developed to deliver multifaceted plant-growth-promoting activities while minimizing plant stress associated with inoculation of a single bacteria species. The seed coatings enabled encapsulation, preservation, and sustained release of plant-growth-promoting bacteria. In addition, they could be stored at room temperature for up to 15 days before use.

In addition, bio-magnetic interfacing has implications in targeted drug delivery and wound healing. The field of bio-magnetic interfacing is long-marked with applications of magnetotactic bacteria (MTB) that contain iron oxide magnetosomes (biomembrane-bound single domain nanocrystals) that enable these bacteria to move along geomagnetic field lines via magnetotaxis^152^. Genetically unaltered MTB has been demonstrated to carry out targeted therapeutic activities such as the killing of *S. aureus* both in vitro and in vivo via magnetic hyperthermia upon application of an alternating magnetic field^153^. Another example involved biohybrid microswimmers, in which motile cells are combined with artificial materials for biosensing or drug delivery applications, composed of MTB MSR-1 and drug-loaded microtubes for targeted antibiotic delivery to *E. coli* biofilms via guidance from an applied rotating magnetic field^154^. While progress in genetic manipulation of MTB has been slow given issues such as cultivation complexity and low native yields of magnetosomes^155,156^, the ability to optimize and control magnetosome production via genetic regulation is crucial for enhancing platform functionality. Progress in this area includes overexpression of magnetosomes in the MTB *Magnetospirillum gryphiswaldense* to control magnetosome size and number^156^, genetically modifying the MTB *Magnetospirillum gryphiswaldense* to overexpress phosphate kinase for enhanced polyphosphate removal from wastewater^157^, as well as the recent development of a toolkit for magnetic nanoparticle multifunctionalization via a tunable magnetosome display system to achieve specific properties^158^. Continued work in this area can ideally lead to the development of highly functional bio-magnetic interfacing systems for deployment in outside-the-lab settings.

### Ongoing challenges and needs

The majority of platforms so far have served as proof-of-concept systems with limited field-deployment. In the case of closed-loop platforms intended for deployment within the human body (engineered probiotics and mammalian cells for pathogenic and non-pathogenic disease treatment) tight control over genetic circuit activation is essential to prevent premature treatment release. Furthermore, implementation of release mechanisms to inactivate devices/engineered cells in the event of complications is essential to assure patient safety (possibly via programmable electronic device integration or use of antibiotic-triggered release mechanisms in the case of mammalian cells^159^). The development of increasingly sophisticated circuits, as demonstrated in proof-of-concept platforms in the above sections, certainly demonstrated progress toward this functionality, but extensive in vivo testing and validation will be required before these technologies can realistically be deployed in humans. Overall, further efforts to engineer organisms least likely to trigger adverse immune responses (such as mammalian cells, probiotics, and even autologous cells from patients) will likely be needed to ensure these treatments are both efficacious and safe.

In terms of external wound healing applications, several of the works described above discuss long-term storage capacity under metabolically inactive conditions. An additional key feature for these systems is the timeline for survival under ambient conditions (exposed to a patient’s skin and the air without access to media) in which wound healing would occur. More detailed knowledge of these timelines could inform further engineering efforts to develop more robust strains as well as yield timelines for swapping of wound dressings in a field setting. Expansion of systems to a broader range of health biomarkers could expand usability as well. For instance, the ingestible bacterial-electronic sensing system described earlier^90^ is an example of an option for the sensing of gut microbiome biomarkers relevant to patient health, which could ideally be coupled with additional ‘response’ circuits to create a closed-loop platform. The possibility for oral administration is also an attractive feature compared to implantation requirements of many other systems.

Within the context of rhizosphere delivery, additional strain engineering efforts are required to further enhance nitrogen fixation and biopesticide activities to outcompete current chemical methods. Furthermore, as these engineered strains are interacting with food products, testing of allergenicity and toxicity of resulting crops will be required to ensure additional allergens and/or toxins are not introduced to the crops via interaction with the engineered microbes.

In terms of biotic/abiotic interfacing, many challenges remain. In particular, extensive biocompatibility testing for all platforms will be required for their successful deployment in humans, in order to identify and eliminate possible adverse immunogenic effects. In the case of implantable devices, fibrosis resulting from the body’s foreign body immune response can impact device efficacy and performance in vivo. This phenomenon requires extensive long-term in vivo testing and can vary with the choice of encapsulation/device material. A potential solution can involve co-delivery of anti-fibrotic drugs with the implanted device, which has been recently demonstrated in vivo in rodents and non-human primates with cells encapsulated in alginate^160^. Furthermore, treatments that require implantation (such as the insulin-resistance and liver disease treatment platforms discussed above) are limited in scope to settings that have at least some degree of hospital access. Integration with delivery mechanisms more conducive to limited-resource settings, such as encapsulation in injectable polymer systems utilizing dynamic covalent bonds^161^ enabling cell-laden gels to transition to a solution phase during injection and reassemble inside the body, could expand applicability to additional outside-the-lab settings.

Biocontainment is another important ongoing issue in the context of delivering engineered cells to humans and agricultural settings. Auxotrophy-based strategies are useful for both limiting the use of antibiotic resistance markers in proximity to other bacteria, as well as potentially confining the survival of engineered hosts to the regions in which they are built to function. Implementation of kill switches, in which cells are programmed to die under specific conditions, can promote increased biocontainment control, but have yet to be demonstrated to have escape frequencies permissible by NIH recommendations^162^. The development of more effective kill switches that are applicable for the organisms used for microbiome/internal drug delivery (probiotics and mammalian cells) is necessary to enable the deployment of these systems. In addition, strain engineering efforts aimed to decrease the potential for genetic variation once deployed in the gut or soil microbiome are necessary to ensure cell-based solutions are safe and efficacious once deployed(strategies for reducing genetic heterogeneity can be found elsewhere^163^). Furthermore, while there is precedent for FDA approval of human cell-based therapies^164^, no live microbial therapeutic has been approved by the FDA (although guidance on their manufacturing and evaluation has been devleoped^165^). Progression from proof-of-concept microbial therapeutic studies to deployment outside-the-lab has many additional challenges including the potential for highly variable microbial behavior in different patient microbiomes, the need for milder large-scale pharmaceutical processes than used for more robust drug products, and potential for loss of microbial viability when traversing the harsh gastric environment^166^.

### Future applications for on-demand outside-the-lab closed-loop delivery platforms

Closed-loop therapeutic delivery systems have the potential to benefit patients and crops in many outside-the-lab scenarios. For instance, continuous drug delivery/disease prevention platforms could be administered to soldiers and astronauts before long, remote military and space missions, respectively in order to decrease the need to obtain or produce needed treatments if a sudden infection arises. In addition, prophylactic treatment of infections could greatly improve patient wellbeing in resource-limited settings such as developing nations, where access to medical resources is scarce. Closed-loop delivery platforms can be used as solutions to lack of patient adherence to medication regimens, a common cause of treatment failure which can be especially prevalent in regions that lack easy access to medical facilities for follow-up visits, as a single administration can enable continuous treatment without risk of medication failure due to inability to follow a dosing regimen. Ideally patients could be administered engineered cellular treatments when at a medical facility, with the closed-loop nature of these treatments decreasing requirements for follow-up visits to hospitals. Some potential administration modes for closed-loop cellular therapies could include smart, wearable bandages to cover and treat wounds or embedded parts of 3D-printed skin grafts. In terms of internal treatments, therapies could be incorporated with polymeric/electronic systems (as discussed earlier in this section) to yield functional living materials. Based on the mode of delivery, these platforms could take the form of cell-laden injectable hydrogels or implantable, drug-eluting stents, as a few potential examples. As a more advanced aspect, these living materials can self-repair or auto-degrade to enable controlled production and release.

In terms of closed-loop plant-growth-promoting systems, platforms could be implemented in remote agricultural regions to promote crop health with limited need for human intervention or administration of chemical fertilizers or pesticides. In the future, stable microbe-based solutions could be applied via aerial application in a similar manner to current crop dusting to maximize efficiency and ease of use. Doing so would require additional research on the long-term stability and safety of microbe-based crop enhancing systems. Beyond terrestrial crops, synthetic biology-based production technologies could be applied to promote terraforming on Mars, in order to modify its current cold, dry, CO_2_-filled environment to make it habitable for Earth-based life forms^167^. Synthetic biology can be used for terraforming approaches for inhabitance by current species^168^ or organisms found on drylands (as the closest case study for Mars’ climate^169^) as well as with genetically modified extremophile organisms with the highest propensity to survive in the current conditions on Mars^170,171^.

## Major ongoing challenges and future directions

While a wide range of scenarios were considered in this Perspective, many outside-the-lab scenarios share common requirements for the development and deployment of synthetic biology platforms. Included in these challenges are synthetically engineering platforms with long-term stability under variable storage conditions, interfacing biology seamlessly with minimal equipment and resource requirements, and minimizing the intervention by experienced professionals through automation and autonomously functioning systems. Enabling these features requires multidisciplinary developments in synthetic biology, materials science, electrical engineering, and other related disciplines to enable outside-the-lab applications. In some cases, currently developed synthetic biology platforms can be (fairly) readily adapted for outside-the-lab use via interfacing with abiotic components, whereas in other cases engineering of new capabilities in both whole-cell and cell-free systems is needed.

Major strategies for maximizing stability for either whole-cell or cell-free approaches have leveraged novel encapsulation of large groups or individual cells and the use of immobilization onto substrates, respectively. The demonstration of lyophilization and subsequent long-term storage potential for many platforms is especially promising for use in remote regions ranging from active military missions to outer space as well as in meeting generalized on-demand conditions. In terms of minimizing equipment and resources, the integration of synthetic biology with platforms such as portable microfluidic devices, wearable hydrogels, and ingestible capsules can better operationalize biotechnology. In doing so, integration and signal detection using simple smartphone apps or even fully automated and/or autonomous performance can minimize the need for operation or intervention by experienced professionals.

As has been discussed, synthetic biology is expanding to applications outside-the-lab. On-demand production of compounds, therapeutics, and materials can obviate the need to stockpile medicines, fuel, or general resources on remote military and space missions. Continuous biosensing of individual health biomarkers and hazards has numerous applications for enhanced healthcare and security in remote regions around and outside the globe. Closed-loop delivery of living therapeutics and engineered probiotics can combat medication noncompliance and antimicrobial resistance, as well as provide sustainable alternatives for chemical fertilizers and pesticides in agricultural regions on Earth and ultimately on Mars and beyond. Reaching these applications will require multidisciplinary work including strategies discussed in detail in the ‘ongoing challenges’ sections described above. Across all of these are needs to stabilize strains for robust performance, enable simple-to-use responsive/autonomous biology, interface biotic-abiotic systems in a seamless manner, and embrace platform simplicity and ease-of-use.

While the recent advancements were organized into application spaces as a way to contextualize their uses, it should be noted that many of the platforms in this Perspective fit multiple categories and thus have transferrable elements that are more broadly applicable. Although many systems were originally designed with a major application in mind, advancements towards enhancing the stability of encapsulated cells or improving multifunctional cell-free assays for example, could yield improvements applicable to any technology aimed towards use in outside-the-lab scenarios. Through continued interdisciplinary innovation, it is possible to develop robust, cost-effective, safe, efficacious platforms that can translate innovative synthetic biology-based technologies developed in the lab into real-world applications in outside-the-lab scenarios. These scenarios can greatly advance many of the grand challenges we face today.
